# Two new species of *Fistulina* (Agaricales, Basidiomycota) from the Northern Hemisphere

**DOI:** 10.3389/fmicb.2022.1063038

**Published:** 2022-12-08

**Authors:** Meng Zhou, Zhan-Bo Liu, Young Woon Lim, Yoonhee Cho, Rui-Heng Yang, Da-Peng Bao, Chang-Lin Zhao, De-Wei Li, Josef Vlasák, Yu-Cheng Dai

**Affiliations:** ^1^School of Ecology and Nature Conservation, Institute of Microbiology, Beijing Forestry University, Beijing, China; ^2^School of Biological Sciences, Institute of Microbiology, Seoul National University, Seoul, South Korea; ^3^Institute of Edible Fungi, Shanghai Academy of Agricultural Sciences, Shanghai, China; ^4^College of Biodiversity Conservation and Utilisation, Southwest Forestry University, Kunming, Yunnan, China; ^5^The Connecticut Agricultural Experiment Station Valley Laboratory, Windsor, CT, United States; ^6^Biology Centre of the Academy of Sciences of the Czechia, České Budějovice, Czechia

**Keywords:** brown rot, Fistulinaceae, polypore, taxonomy, wood-decaying fungi

## Abstract

Phylogenetic and morphological analyses on samples of *Fistulina* from East Asia and North America were carried out, and two new species were described, namely, *Fistulina americana* and *Fistulina orientalis*, both previously known as *Fistulina hepatica*. The former is characterized by lateral stipitate basidiocarps, relatively small pores (7–8 per mm), a monomitic hyphal system with both clamp connections and simple septa, and ellipsoid basidiospores of 4–4.8 × 3–3.3 μm, and the species has been found on *Quercus* in North-East USA. *F. orientalis* is characterized by lateral stipitate basidiocarps, very small pores (11–12 per mm) with pruinose dissepiments, a monomitic hyphal system with both clamp connections and simple septa, and ovoid to subglobose basidiospores of 3–4 × 2.7–3 μm, and the species has been found on *Castanopsis* in East Asia. Phylogenetically, samples of *F. americana* and *F. orientalis* form two new lineages nested in the *Fistulina* clade.

## Introduction

*Fistulina* Bull. was established by Bulliard (1791) and typified by *Fistulina hepatica* (Schaeff.) With. The genus is characterized by annual, pileate to lateral stipitate basidiocarps with reddish to brownish upper surface and context with red sap when fresh, separated tubes closely packed, a monomitic hyphal system with clamp connections, some with simple septa, cystidial elements present at dissepimental edges, hyaline, thin- to thick-walled basidiospores that are cyanophilous, and the degradation of hardwoods as a brown rot (Ryvarden and Melo, 2017). It is a cosmopolitan genus with ten species accepted, eight from the Southern Hemisphere and two from the Northern Hemisphere (González et al., 2021). Although *Fistulina* is considered a polypore genus, it consists of separate tubes, which is a feature different from the real polypores. Phylogenetically, *Fistulina* is closely related to *Porodisculus* Murrill in the euagarics clade (Bodensteiner et al., 2004; Binder et al., 2005; Song et al., 2015; Sun et al., 2019; González et al., 2021).

*Fistulina hepatica* is known as a tongue mushroom or beefsteak polypore because the juvenile fruiting body resembles a huge tongue in pinkish-red color and exudes a reddish blood-like sap when squeezed or bruised (Ryvarden and Gilbertson, 1993). The distinct morphological characteristics make it easy to identify *F. hepatica*, which has been recorded as a common species in Europe, North America, and North Asia (Gilbertson and Ryvarden, 1986; Núñez and Ryvarden, 2001; Ryvarden and Melo, 2017). However, a recent study showed that the taxon from Southwest China was different from the real *F. hepatica*, and the species, *Fistulina subhepatica* B.K. Cui and J. Song was described as new (Song et al., 2015). As the type specimen of *F. hepatica* was collected in Europe, there is a high probability that specimens collected in other distant regions could correspond to different species. In some wood decay fungi, it is common to treat the geographical distribution as an important indicator to distinguish species. Asian *Ganoderma lucidum* was proposed to *Ganoderma lingzhi* (Cao et al., 2012) and the cosmopolitan polypore *Laetiporus sulphureus* was separated into several species by continents (Vasaitis et al., 2009; Song et al., 2018).

Previously, Gilbertson and Ryvarden (1986) and González et al. (2021) treated North American *Fistulina* as *F. hepatica*. In this study, samples from East Asia and North America were analyzed. Molecular phylogeny based on a combined ITS and nLSU dataset revealed two new independent lineages. In addition, morphological differences between the two new species from *F. hepatica* are distinct. Detailed descriptions of the two new species are reported.

## Materials and methods

### Morphological studies

The studied specimens are deposited in the herbaria of Beijing Forestry University (BJFC), Southwest Forestry University (SWFC), the Connecticut Agricultural Experiment Station Valley Laboratory (NHES), and Seoul National University Fungus Collection (SFC). Macro-morphological descriptions were based on field notes and voucher herbarium specimens. Microscopic measurements and drawings were made from slides prepared from voucher tissues and stained with Cotton Blue and Melzer’s reagent. The following abbreviations were used: KOH = 5% potassium hydroxide; CB = Cotton Blue; CB+ = cyanophilous in Cotton Blue; CB– = acyanophilous in Cotton Blue; IKI = Melzer’s reagent; IKI– = neither amyloid nor dextrinoid in Melzer’s reagent; *L* = mean basidiospore length (arithmetic average of basidiospores); *W* = mean basidiospore width (arithmetic average of basidiospores); *Q* = variation in the L/W ratios between specimens studied; *n* (a/b) = number of basidiospores (a) measured from the given number of specimens (b). In presenting basidiospore size variation, 5% of measurements were excluded from each end of the range and these values are given in parenthesis. Special color terms follow Anonymous (1969) and Petersen (1996).

### DNA extraction, amplification, and sequencing

A CTAB rapid plant genome extraction kit-DN14 (Aidlab Biotechnologies Co., Ltd., Beijing, China), AccuPrep Genomic DNA Extraction Kit (Bioneer, Daejeon, Korea), and FH plant DNA kit II (Demeter Biotech Co., Ltd., Beijing, China) were used to extract total genomic DNA from dried specimens and to perform the polymerase chain reaction (PCR) according to the manufacturer’s instructions with some modifications (Shen et al., 2019). The ITS region was amplified with primer pairs ITS5 (GGA AGT AAA AGT CGT AAC AAG G) and ITS4 (TCCTCC GCT TAT TGA TAT GC) (White et al., 1990), and for nLSU, LR0R (ACC CGC TGA ACT TAA GC), and LR7 (TAC TAC CAC CAA GAT CT) (Vilgalys and Hester, 1990). The final PCR volume was 30 μl; each tube contained 1 μl of each primer, 1 μl extracted DNA, 12 μl ddH_2_O, and 15 μl 2 × EasyTaq PCR Supermix (TransGen Biotech Co., Ltd., Beijing, China). PCRs were performed on S1000™ Thermal Cycler (Bio-Rad Laboratories, Hercules, CA, USA). The PCR procedure for ITS was as follows: initial denaturation at 95°C for 3 min, followed by 34 cycles of denaturation at 94°C for 40 s, annealing at 54°C for 45 s, and extension at 72°C for 1 min, followed by the final extension at 72°C for 10 min. The PCR procedure for‘ nLSU was initial denaturation at 94°C for 1 min, followed by 34 cycles of denaturation at 94°C for 30 s, annealing at 50°C for 1 min, and extension at 72°C for 1.5 min, followed by the final extension at 72°C for 10 min. The PCR products were purified and sequenced at the Beijing Genomics Institute (BGI), China, using PCR primers. All sequences analyzed in this study were deposited at GenBank and listed in Table 1.

**TABLE 1:** Information for the sequences used in this study.

Species	Specimen	Location	Host	GenBank accession no.	
				
				ITS	nLSU
***Fistulina americana***	**CLZhao 147**	**Massachusetts, USA**	***Quercus* sp.**	**MG231510**	–
***Fistulina americana***	**DL-22-189**	**Massachusetts, USA**	***Quercus* sp.**	**OP806861**	**OP806860**
***Fistulina americana***	**REG593**	**USA**	Unknown	**AY571038**	**AY571004**
*Fistulina antarctica*	1015	Argentina	*Nothofagus antarctica*	MW462921	MW462925
*Fistulina antarctica*	CBS701.85	Argentina	Unknown	DQ486702	AY293181
*Fistulina endoxantha*	GM19079	Argentina	*Lophozonia obliqua*	MW462919	MW462956
*Fistulina endoxantha*	GM19089	Argentina	*Lophozonia alpina*	MW462920	MW462957
*Fistulina hepatica*	CCBAS532	Czech Republic	Unknown	LN714544	–
*Fistulina hepatica*	FCL <POL> : 457	Poland	Unknown	KY474052	–
***Fistulina orientalis***	**SFC20210518-01**	**Jeju Island, Korea**	***Castanopsis sieboldii***	**OP595749**	**OP595747**
***Fistulina orientalis***	**YRH 217**	**Anhui, China**	***Castanopsis eyrei***	**OP595750**	**OP595748**
*Fistulina pumiliae*	GM19077	Argentina	*Nothofagus pumilio*	MW462917	MW462954
*Fistulina pumiliae*	GM19078	Argentina	*Nothofagus pumilio*	MW462918	MW462955
*Fistulina pumiliae*	GM19018	Argentina	*Nothofagus pumilio*	MW462914	MW462953
*Fistulina subhepatica*	Cui 11130	Yunnan, China	Angiosperm	KJ925059	KJ925054
*Fistulina subhepatica*	Dai 13216	Yunnan, China	*Castanopsis* sp.	KJ925060	KJ925055
*Fistulina tasmanica*	Cui 16605	Tasmania, Australia	*Eucalyptus* sp.	MK986821	MK986823
*Fistulina tasmanica*	Cui 16635	Tasmania, Australia	*Eucalyptus* sp.	MK986822	MK986824
*Porodisculus pendulus*	HUO12158	Colombia	Unknown	EU423190	–
*Porodisculus pendulus*	HHB13576	Wisconsin, USA	Unknown	KX065960	KX065994
*Pseudofistulina radicata*	G1080	USA	Unknown	–	MK278531
*Pseudofistulina radicata*	CBS 508.63	USA	Unknown	AY571039	AY571005

New taxa are in bold.

### Phylogenetic analyses

Phylogenetic trees were constructed using ITS + nLSU rDNA sequences, and phylogenetic analyses were performed with the maximum likelihood (ML) and Bayesian inference (BI) methods. Sequences of the species and strains were primarily adopted from ITS-based and 28S-based tree topology, as described by González et al. (2021). New sequences generated in this study, along with reference sequences retrieved from GenBank (Table 1), were aligned by MAFFT version 7 (Katoh et al., 2019^1^) using the “G-INS-i” strategy and manually adjusted in BioEdit (Hall, 1999). Unreliably aligned sections were removed before the analyses, and efforts were made to manually inspect and improve the alignment. The data matrix was edited in Mesquite version 3.70 (Maddison and Maddison, 2021). The sequence alignment was deposited at TreeBase (submission ID 29857). Sequences of *Pseudofistulina radicata* (Schwein.) Burds. obtained from GenBank were used as outgroups to root the trees in the ITS + nLSU analysis.

The research using ML was conducted using RAxML-HPC version 8.2.3 (Stamatakis, 2014) and RAxML-HPC through the CIPRES Science Gateway (Miller et al., 2009^2^). Statistical support values (BS) were obtained using non-parametric bootstrapping with 1,000 replicates.

jModelTest version 2.17 was used to determine the best-fit evolution model of the combined dataset for ML and BI (Darriba et al., 2012). Four unique partitions were established, and GTR + I + G was a selected substitution model for each partition. The BI was calculated with MrBayes version 3.2.6 (Ronquist et al., 2012) in two independent runs, each of which had four chains for 2 million generations and started from random trees. Trees were sampled every 100 generations. The first 25% of sampled trees were discarded as burn-in, whereas other trees were used to construct a 50% majority consensus tree and for calculating Bayesian posterior probabilities (BPPs).

Phylogenetic trees were visualized using FigTree version 1.4.4 (Rambaut, 2018). Branches that received bootstrap support (BS) for ML and BPPs ≥ 75% (BS) and 0.95 (BPP) were considered significantly supported, respectively.

## Results

### Phylogeny

**FIGURE 1:**
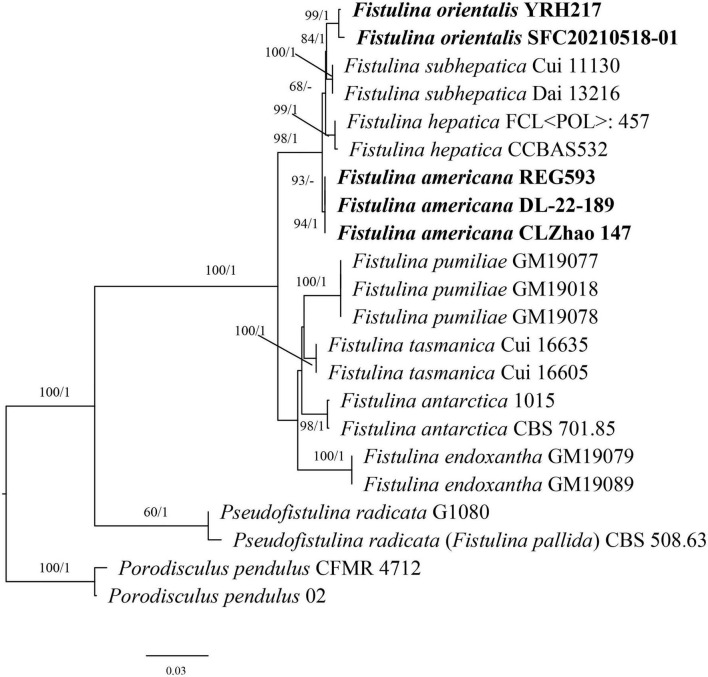
Maximum likelihood analysis of *Fistulina* based on the dataset of ITS + nLSU. The bootstrap values higher than 50% and BPPs values more than 0.90 are shown. New species are mentioned in bold.

The ITS + nLSU dataset contained sequences from 22 fungal specimens, representing eight species of *Fistulina*, *Porodisculus pendulus*, and *P. radicata*; it had an aligned length of 1,977 characters. The Bayesian analyses exported a nearly identical topology to the ML analyses with an average standard deviation of split frequencies = 0.007082. Therefore, only the ML tree is presented with the BS and BPP. The phylogeny (Figure 1) inferred from the ITS and nLSU sequences showed that the sequences of *Fistulina americana* sp. nov. and *Fistulina orientalis* sp. nov. nested in the *Fistulina* clade and formed two independent lineages; both new species are related to *F. hepatica* and *F. subhepatica* with strong support (98% BS, 1 BPP).

### Taxonomy

**FIGURE 2:**
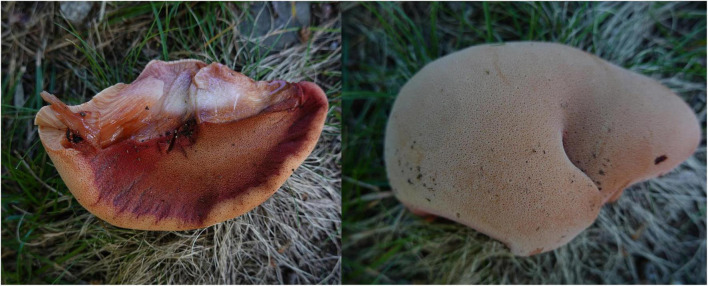
Basidiocarps of *Fistulina americana* (DL-22-189).

**FIGURE 3:**
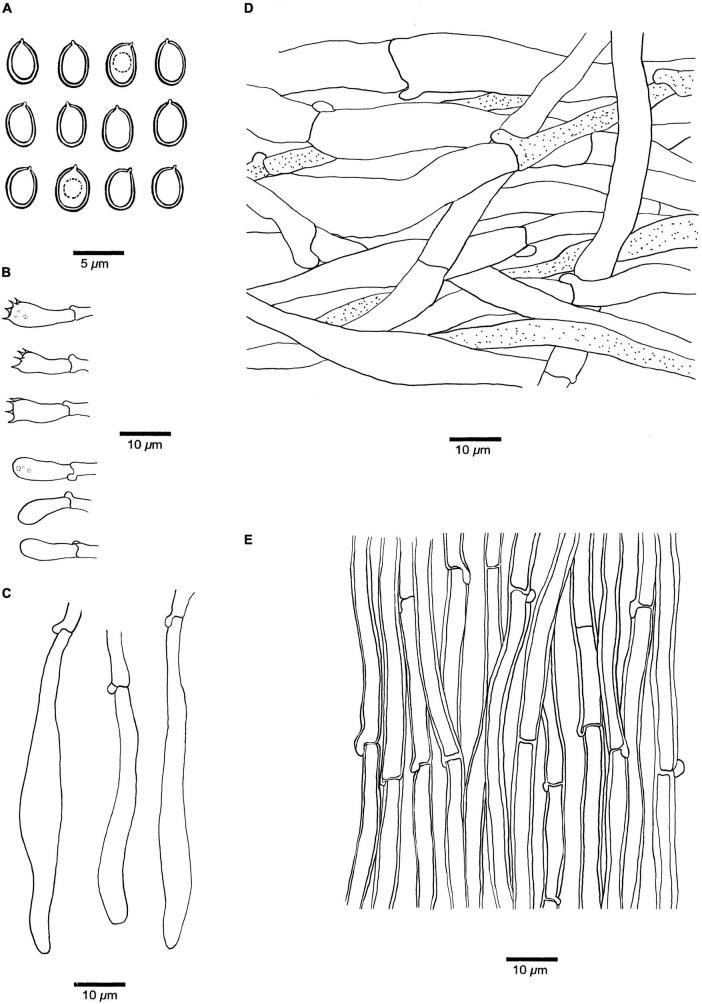
Microscopic structures of *Fistulina americana* (drawn from the holotype, BJFC038583). **(A)** Basidiospores. **(B)** Basidia and basidioles. **(C)** Cystidial elements at dissepimental edges. **(D)** Hyphae from context. **(E)** Hyphae from tube trama.

***Fistulina americana*** Y.C. Dai, D.W. Li, and Meng Zhou, sp. nov. Figures 2, 3.

MycoBank: **MB 846428**.

Differs from other *Fistulina* species by ellipsoid basidiospores 4–4.8 × 3–3.3 μm, and growth on *Quercus* in North-East USA.

**Type**. USA, Massachusetts, Boston, Blackstone Square Park, 42°20′23.4″N, 71°04′24.8″W, on the stump of *Quercus*, 27.VII.2015, C.L. Zhao 147 (holotype, SWFC 000147; isotype, BJFC038583).

**Etymology**. *Americana* (Lat.): refers to North America, where the species was found.

**Basidiomata.** Annual, lateral stipitate, fleshy, and readily exuding a reddish blood-like sap when squeezed or bruised when fresh, hard corky when dry. Pileus dimidiate to fan-shaped, projecting up to 6 cm, 5 cm wide, and 8 mm thick at the base when dry. Pileal surface pinkish brown to reddish brown, slimy, radially striate when fresh, becoming cinnamon to vinaceous gray, irregularly zonate when dry; margin blunt, concolorous with pileal surface or paler than pileal surface. Pore surface white to flesh-pink when fresh, become brownish when bruised, fulvous to umber when dry, bruised part become black when dry; sterile margin almost absent; pores round, 7–8 per mm, consisting of individual, crowed but easily separable tubes; dissepiments thin, usually entire, slightly pruinose. Context pale mouse gray and corky when dry, up to 5 mm thick. Tubes peach, paler than pore surface, slightly rigid when dry, up to 3 mm long. Stipe concolorous with pileal surface when fresh and dry, up to 13 mm long and 10 mm in diameter.

**Hyphal structure.** Hyphal system monomitic; generative hyphae with clamp connections and simple septa, IKI–, CB– to slightly CB+, become swollen in KOH.

**Context.** Generative hyphae hyaline, thin-walled, occasionally branched, interwoven, some collapsed, 7–11 μm in diameter, some inflated up to 22 μm in diameter; gloeoplerous hyphae present.

**Tubes.** Generative hyphae hyaline, thin- to slightly thick-walled, rarely branched, gelatinous, parallel along the tubes, 5–7 μm in diameter. Basidia clavate with four sterigmata and a basal clamp connection, 19–25 × 4.5–7 μm; basidioles in shape similar to basidia, but slightly smaller. Cystidial elements present at dissepimental edges, hyaline, thin-walled, smooth, with an oily substance, 74–87 × 7–9 μm.

**Spores.** Basidiospores ellipsoid, hyaline, thick-walled, smooth, with a big guttule, IKI–, CB+, (3.8–)4–4.8(–5) × (2.8–)3–3.3(–3.5) μm, *L* = 4.18 μm, *W* = 3.06 μm, *Q* = 1.37 (*n* = 30/1).

*Additional specimens examined.* USA. Connecticut, Avon, Avon Old Farm School, on the fallen trunk of *Quercus palustris*, 24.IX.2022, DL-22-192 (NHES, Dupl. BJFC); East Hampton, Hurd State Park, on the stump of *Quercus*, 4.IX.2022, DL-22-189 (NHES, dupl. in BJFC and JV); West Harford, 6 Reservoir, on stump of *Quercus*, 10.IX.2021, DL-21-209 (NHES, dupl. in BJFC and JV).

**FIGURE 4:**
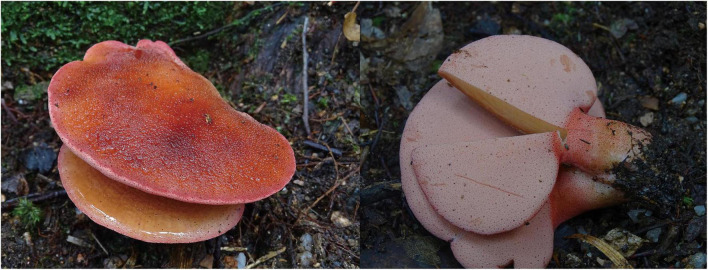
Basidiocarps of *Fistulina orientalis* (holotype, BJFC038584).

**FIGURE 5:**
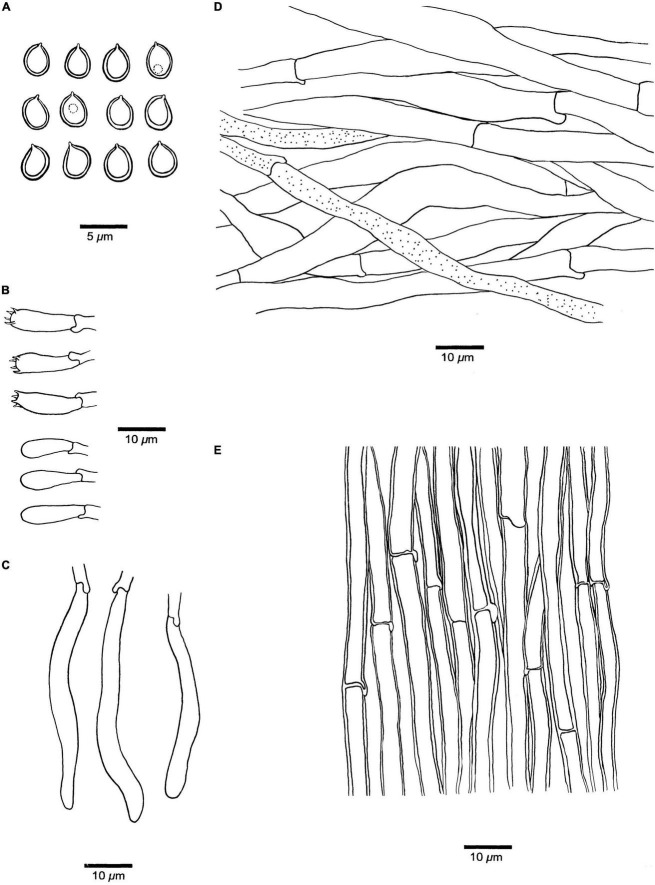
Microscopic structures of *Fistulina orientalis* (drawn from the holotype BJFC038584). **(A)** Basidiospores. **(B)** Basidia and basidioles. **(C)** Cystidial elements at dissepimental edges. **(D)** Hyphae from context. **(E)** Hyphae from tube trama.

***Fistulina orientalis*** Y.C. Dai, D.P. Bao, and Y.W. Lim, sp. nov. Figures 4, 5.

MycoBank: MB 846430.

Differs from other *Fistulina* species by its small pores of 11–12 per mm, small basidiospores of 3–4 × 2.7–3 μm, and growth on *Castanopsis* in East Asia.

**Type.** China, Anhui Province, Huangshan County, Huangshan Forest Park, Diaoqiao, on the living tree of *Castanopsis eyrei*, alt. 500 m, 30°08′35.44″ N, 118°06′45.59″ E, 7.IX.2018, R.H. Yang 217 (holotype, BJFC038584).

**Etymology.** *Orientalis* (Lat.): refers to East Asia where the species was found.

**Basidiomata**. Annual, lateral stipitate, fleshy and readily exuding a reddish blood-like sap when squeezed or bruised when fresh, woody hard to bone hard when dry. Pileus dimidiate to fan-shaped, projecting up to 4 cm, 5 cm wide, and 6 mm thick at the base when dry. Pileal surface salmon to scarlet, slimy, and faintly radially furrowed when fresh, becoming black to blackish blue and irregularly zonate upon drying; margin acute, concolorous with pileal surface. Pore surface flesh-pink when fresh, become brown when bruised, clay buff when dry, bruised part become black when dry; sterile margin almost absent; pores round, 11–12 per mm, consisting of individual, crowed but easily separable tubes; dissepiments thick, entire, pruinose. Context cream when fresh, dark gray and bone hard when dry, up to 4 mm thick. Tubes concolorous with pore surface, rigid when dry, and up to 2 mm long. Stipe concolorous with pileal surface when fresh, become dark gray when dry, up to 25 mm long and 5 mm in diameter.

**Hyphal structure.** Hyphal system monomitic; generative hyphae with clamp connections and simple septa, IKI–, CB–, become swollen in KOH.

**Context**. Generative hyphae hyaline to pale brownish, thin-walled, occasionally branched, interwoven, some collapsed, 6–10 μm in diameter, gloeoplerous hyphae present.

**Tubes**. Generative hyphae hyaline, thin- to slightly thick-walled, rarely branched, gelatinous, parallel along the tubes, 3–6 μm in diameter. Basidia clavate with four sterigmata and a basal clamp connection, 19–22 × 5–7 μm; basidioles in shape similar to basidia, but slightly smaller. Cystidial elements present at dissepimental edges, hyaline, smooth, thin-walled, with an oily substance, 57–85 × 5–7 μm.

**Spores**. Basidiospores ovoid to subglobose, hyaline, thick-walled, smooth, IKI–, CB+, (2.9–)3–4(–4.1) × (2.6–)2.7–3(–3.2) μm, *L* = 3.36 μm, *W* = 2.93 μm, *Q* = 1.15 (*n* = 30/1).

*Additional specimen examined*. Korea. Jeju Island, Seogwipo-si Gosali Forest Road, on dead root of living *Castanopsis sieboldii*, alt. 300 m, 33°31′63.86″ N, 126°59′76.26″ E, 18.V.2021, H.T. Jang (SFC20210518-01).

## Discussion

By inclusion of the two new species we have described here, twelve species are now recognized in *Fistulina*. Among them, eight species have a distribution in the Southern Hemisphere (González et al., 2021), while the remaining four species, *F. americana*, *F. hepatica*, *F. orientalis*, and *F. subhepatica*, are found in the Northern Hemisphere (Song et al., 2015; Ryvarden and Melo, 2017). The four Northern Hemisphere species are closely related to our phylogenetic analysis (Figure 1). *F. americana* has been considered as the European *F. hepatica* (Gilbertson and Ryvarden, 1986; Wu et al., 2022). However, according to the phylogenetic analysis, our specimens together with one specimen from GenBank (REG593 from the USA, Bodensteiner et al., 2004) formed an independent lineage with strong support (94% BS, 1 BPP). In addition, there is more than 11-base-pair difference between the sequences of *F. americana* and *F. hepatica*, which accounts for >1.5% of the nucleotides in the ITS regions. Morphologically, *F. americana* can be differentiated from *F. hepatica* by smaller pores (7–8 vs. 2–5 per mm, Niemelä, 2016) and narrower basidiospores (ellipsoid and 3–3.3 μm wide vs. ovoid to tear-shaped and 3.3–4.3 μm wide, Niemelä, 2016). *F. americana* resembles *F. subhepatica* in sharing the nearly same size of pores and basidiospore dimension, but the latter species has clamp connections without simple septa, while the former has both the clamp connections and simple septa on generative hyphae. In addition, *F. americana* grows on *Quercus* and has a limited distribution in North America, while *F. subhepatica* is found on *Lithocarpus* and is distributed in Southwest China. Meanwhile, the nucleotide difference between *F. americana* and *F. subhepatica* sequences was more than 1.5% in the ITS regions.

Molecularly, there is more than 2% of nucleotide difference between the sequence of *F. americana* and *F. orientalis* in the ITS regions. Morphologically, *F. orientalis* is readily distinguished from all other *Fistulina* species by its smaller pores (11–12 per mm) and smaller basidiospores measuring 3–4 × 2.7–3 μm (pores < 10 per mm and basidiospores >4 μm long in other species, González et al., 2021).

## Data availability statement

The datasets presented in this study can be found in online repositories. The names of the repository/repositories and accession number(s) can be found in the article/supplementary material.

## Author contributions

MZ, Z-BL, YC, and Y-CD coordinated the project and designed the experimental plan. MZ and Z-BL analyzed the data with help from C-LZ, JV, R-HY, YC, and YL. C-LZ, D-PB, D-WL, R-HY, and YL collected the samples from the field. MZ and Y-CD wrote the original draft preparation. MZ, Y-CD, D-WL, JV, YL, and YC reviewed and edited the manuscript. Y-CD and YL acquired funding. All authors contributed to the article and approved the submitted version.

## Footnotes

1
http://mafft.cbrc.jp/alignment/server/

2
http://www.phylo.org
